# Micro/nanorobots in antimicrobial therapy: Addressing challenges of antibiotic resistance

**DOI:** 10.1016/j.mtbio.2025.101936

**Published:** 2025-05-31

**Authors:** Xutong Chen, Yong Li, Chunhua Wang, Zhiqiang Chen, Zhijie Xu, Fada Xia, Yuanliang Yan, Ming Gao

**Affiliations:** a State Key Laboratory of Environmental Chemistry and Ecotoxicology, Research Center for Eco-Environmental Sciences, Chinese Academy of Sciences, Beijing, China; b University of Chinese Academy of Sciences, Beijing, China; c Department of general Surgery, Turpan City People's Hospital, Tulufan, Xinjiang, China; d Department of Pediatric Surgery, Hunan Children's Hospital, Changsha, Hunan, China; e Department of Oral Bioscience, Tokushima University Graduate School of Biomedical Sciences, Tokushima, Japan; f Department of Pathology, Xiangya Hospital, Central South University, Changsha, Hunan, China; g Department of Thyroid Surgery, National Clinical Research Center for Geriatric Disorders, Xiangya Hospital, Central South University, Changsha, Hunan, China; h Department of Pharmacy, Xiangya Hospital, Central South University, Changsha, Hunan, China

**Keywords:** Micro/nano robots, Biofilms, Bacterial infection, Antibacterial therapy

## Abstract

Antibiotic resistance has emerged as a critical global health challenge, particularly when bacteria form biofilms that render conventional antimicrobial treatments markedly less effective. Bacteria residing within biofilm exhibit increased resistance to antimicrobial agents and host immune defenses, complicating treatment and contributing to recurrent infections. Antimicrobial micro- and nanorobots (MNRs) have garnered significant attention as a promising strategy to combat drug-resistant bacteria and biofilms, owing to their exceptional motility, precise targeting, and improved penetration capabilities. Despite their potential, challenges related to biocompatibility, imaging integration, and clinical translation remain unresolved. This review summarizes the latest developments in the therapy of micro/nanorobots for antimicrobial therapy, emphasizing innovative strategies for bacterial eradication and biofilm disruption while addressing the technical hurdles and exploring future research directions.

## Graphical abstract

**Figure:**
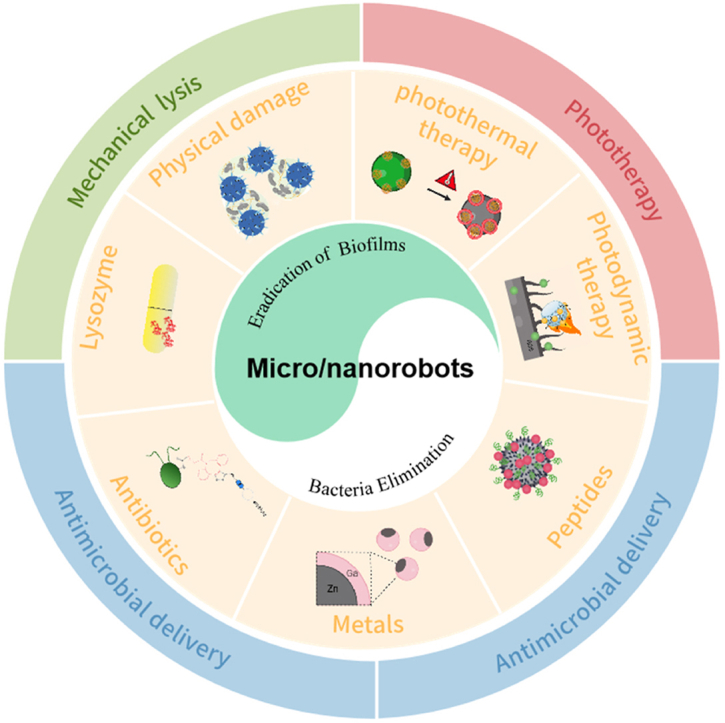

## 1 Introduction

Bacterial infections, particularly those associated with antibiotic resistance and biofilms, pose significant challenges to global health. The misuse and over-reliance on antibiotics have contributed to the growing problem of bacterial resistance, particularly in diseases with frequent and complicated infections. The emergence of drug-resistant bacteria has rendered traditional treatments increasingly ineffective [[1], [2], [3], [4]]. The World Health Organization (WHO) estimates that antimicrobial resistance results in roughly 700,000 global fatalities each year, with projections suggesting this number could escalate to 10 million by 2050—surpassing cancer-related mortality—if effective countermeasures are not implemented [5,6]. The increase in bacterial resistance is closely related to biofilms [7,8]. Biofilms are complex three-dimensional structures formed by bacteria on host surfaces, enabling their survival in diverse environments and rendering them highly resistant to antibiotics and immune responses [[9], [10], [11]]. Their presence often limits the effectiveness of antibiotics at the core of infection sites, leading to persistent infections and frequent recurrence [12]. Thus, effectively eliminating drug-resistant bacteria and eradicating biofilms have remained key challenges in antimicrobial therapy research.

Micro/nanorobots (MNRs), also referred to as micro/nanomotors or micro/nanoswimmers [13,14], have demonstrated unique advantages under the limitations of conventional therapeutic approaches, owing to their autonomous motility, intelligent responsiveness, and multi-mechanism synergy [15]. The propulsion mode of MNRs forms the basis for their functional performance. Currently, the propulsion of MNRs can be broadly classified into two major categories: chemical reaction-driven and externally powered systems [16]. Chemically driven MNRs generate motion through catalytic reactions; for instance, enzyme-powered MNRs can achieve in situ fuel supply within biological environments, offering excellent biocompatibility. Externally powered MNRs, such as those driven by magnetic fields, light, or ultrasound, enable precise control with multiple degrees of freedom (DOF) and allow for noninvasive localization and navigation without relying on chemical fuels.

Compared to passive diffusion-based nanomedicines, MNRs can achieve targeted delivery through external field propulsion (e.g., magnetic fields, ultrasound, light) or chemical self-propulsion (e.g., catalytic reactions), actively penetrating dense biofilms or mucus layers and enhancing drug accumulation at infection sites. Furthermore, their intelligent responsiveness—triggered by stimuli such as pH, hydrogen peroxide (H_2_O_2_), or enzymatic signals—allows real-time sensing of the infectious microenvironment and on-demand drug release, thereby reducing off-target toxicity. In addition, MNRs can integrate mechanical disruption (e.g., physical destruction of biofilms) with chemical bactericidal mechanisms (e.g., catalytic generation of reactive oxygen species), leading to enhanced antibacterial efficacy [[17], [18], [19]]. Table 1 summarizes the key advantages of different antimicrobial strategies employed by MNRs for bacterial and biofilm eradication [[20], [21], [22], [23]]. Overall, the dynamic delivery, environmental responsiveness, and multimodal antibacterial actions of MNRs offer a promising approach for addressing challenges in bacterial infection treatment, particularly in the context of biofilm-associated and drug-resistant infections.

**Table 1:** Key advantages of micro/nanorobots in bacterial and biofilm eradication.

Antibacterial Strategy	Mechanism of Action	Key Advantages	Refs
Antibacterial-loaded	Targeted delivery of antibiotics, metal ions, lysozymes, or antimicrobial peptides	Enhances local concentration, decreases the required dosage, and reduces the probability of resistance development	20
ROS systems	Catalytic, light- or ultrasound-triggered ROS generation	Damages bacterial membranes and biofilms; strong and broad-spectrum sterilization effect	21
Physical destruction	Active movement or vibration breaks the biofilm matrix	Does not propagate antimicrobial resistance; remote controllability	22
Synergistic action	Combination of physical disruption, chemical killing, and ROS generation	Offers superior efficacy, deeper penetration, and minimized resistance	23

In this review, we provide a concise overview of the design principles, material choices, and propulsion strategies of antimicrobial MNRs, with an emphasis on magnetic-driven, light-driven, enzyme-driven, and combined propulsion systems. We then systematically examine recent advances in their application for bacterial clearance and biofilm disruption. Achievements and challenges related to biocompatibility and imaging are discussed, followed by an exploration of strategies to overcome technical and biological barriers toward clinical translation. Several existing reviews have systematically summarized the progress of MNRs in antimicrobial therapy, covering propulsion mechanisms, structural design, material construction, and their applications in bacterial elimination and biofilm disruption. However, most of these reviews primarily focus on functional exploration and technical classification at the experimental level. In contrast, this review builds upon current therapeutic strategies and antibacterial mechanisms, while placing greater emphasis on the key challenges faced in clinical translation, including biocompatibility, biodegradability, safety evaluation, precise control, and imaging guidance. Moreover, we systematically highlight recent integrated strategies such as stimuli-responsive therapies, theranostic integration, and immune-evasive surface modifications, which have received limited attention in prior reviews. This work aims not only to complement existing literature but also to offer a more application-oriented perspective that may serve as a reference for future research and potential clinical translation of antimicrobial MNRs.

## 2 Design and propulsion mechanism of micro/nanorobots

Micro/nanorobots are intelligent, responsive systems capable of autonomous or externally controlled motion at the microscale. Their design typically follows a structure–function coupling paradigm: by tuning geometric architecture, material composition, and surface functionalization, multidimensional control and bioresponsiveness can be achieved. Notably, material selection must balance propulsive efficiency and biocompatibility; inorganic materials such as magnetic nanoparticles (Fe_3_O_4_, Co), photoresponsive semiconductors (TiO_2_, BiVO_4_), and noble metals (Ag, Au) are commonly employed to construct the propulsive core, whereas polymers (e.g., PLGA, PEG), carbon‐based materials (e.g., graphene oxide), and natural biomolecules are used to enhance drug-loading capacity and biological stealth. Propulsion mechanisms—central to robot functionality—can be broadly classified by energy source and actuation mechanism into exogenous physical drives, chemical drives, and combined drives. In this section, we focus on magnetic and light-driven among exogenous physical drives, enzyme-driven among chemical drives, and combined propulsion.

### 2.1 Magnetic actuated

Magnetic propulsion is a mature and efficient exogenous physical driving strategy. By incorporating magnetic materials, such as Fe_3_O_4_ nanoparticles, nickel, or cobalt, into the structure of MNRs, precise navigation within biological environments can be achieved. Using rotating, oscillating, or gradient magnetic fields, these robots can exhibit a variety of motion modes, including rolling, helical propulsion, and swinging. Magnetic actuation does not require chemical fuel and avoids cytotoxicity; commonly used ferromagnetic microrobots have demonstrated excellent biocompatibility and recyclability. Owing to their controllable motion, magnetically driven MNRs can be directed toward otherwise inaccessible regions, enabling deep tissue penetration and targeted therapy. Moreover, magnetic MNRs can be coupled with magnetic hyperthermia to enhance bactericidal efficacy by disrupting bacterial membranes under elevated temperatures. Bhuyan et al. designed biocompatible micromotors (T-Budbots) derived from tea buds, which could be magnetically guided into biofilm matrices to achieve precise disruption and removal [24]. In 2019, Liu et al. reported magnetically active copper ferrite nanoparticles and demonstrated their antibacterial activity in an in vivo subcutaneous abscess model [25]. Notably, the pronounced magnetism of copper ferrite nanoparticles enabled a 20-fold enhancement of photothermal effects via magnetic enrichment, facilitating highly effective sterilization at ultra-low doses and improving biosafety.

However, despite substantial progress in experimental studies, several challenges remain for magnetic propulsion. For instance, in scenarios involving multiple robots operating simultaneously, magnetic field interference and inter-robot coupling may lead to unpredictable motion behaviors. In addition, most current magnetic control systems rely on bulky external equipment, limiting their clinical applicability. Therefore, simplifying magnetic field generation devices and enhancing the real-time responsiveness of robot manipulation are critical for advancing this technology toward practical biomedical applications.

### 2.2 Light driven

Light-driven represents another commonly used exogenous physical driving strategy, which relies on photoresponsive materials capable of absorbing and converting specific wavelengths of light via mechanisms such as photothermal and photocatalytic effects. Typical materials include gold nanorods, BiVO_4_, and TiO_2_, which, under laser or LED illumination, can generate temperature, gas, or ion gradients to drive the directional motion of MNRs. Light-driven systems offer submicron-level spatial precision, making them well-suited for targeted therapies in superficial tissues, such as skin wounds or oral infections. Meanwhile, photothermal or photocatalytic reactions can also induce the production of bactericidal agents such as reactive oxygen species (ROS), forming a combined “light-driven + antibacterial” strategy. In 2019, Pumera's group developed visible-light-driven BiVO_4_ micromotors, which were used for the selective capture and elimination of microorganisms [26]. Under optical control, these micromotors were capable of translating along linear trajectories on various axes and showed high-affinity adhesion to live microbes. Moreover, the team led by Tijana reported a near-infrared light-driven mesoporous SiO_2_/Au nanomotor, which exhibited strong self-propulsion due to the photothermal effect of the gold component [27]. This allowed the nanomotor to penetrate and disrupt biofilm matrices, achieving significant eradication of *Pseudomonas aeruginosa* biofilms even with short irradiation times (30s–3 min). It should be noted, however, that a major limitation of light-driven propulsion lies in the limited tissue penetration depth of light, posing a challenge for clinical applications that require the delivery of optical energy to deep-seated tissues.

### 2.3 Enzyme driven

Enzyme-driven represents an endogenous fuel–based actuation mode for MNRs, offering a more physiologically relevant driving mechanism compared to exogenous chemical fuels. These robots are typically functionalized with enzymes such as urease, peroxidase, or glucose oxidase, which catalyze specific substrates to generate gas (e.g., O_2_ or NH_3_) or ion flows, thereby driving local microflows or self-propulsion. For instance, urease-catalyzed hydrolysis of urea in a urinary tract infection milieu produces gas bubbles that propel the robot penetrating the biofilm, enabling precise localization of the infection site. In 2021, Xu et al. reported a urease-powered liquid-metal (LM) nanorobot: surface-anchored urease endowed the LM robot with chemotactic transport along urea concentration gradients, while NIR irradiation induced shape deformation of the LM core, conferring enhanced photothermal antibacterial activity [28]. Enzyme-driven systems feature strong microenvironment adaptability, efficient utilization of endogenous substrates, and excellent biocompatibility, making them especially suited to metabolically abnormal or substrate-rich pathological sites. Nevertheless, issues such as enzyme stability, risk of inactivation, and potential toxicity of byproducts remain to be addressed.

### 2.4 Combined propulsion

Combined propulsion has emerged as a key strategy in micro/nanorobot research by integrating two or more distinct driving mechanisms to combine their complementary advantages and overcome the limitations of single-mode actuation in complex environments. By incorporating multifunctional materials or multi-responsive modules at the structural design level, hybrid robots achieve superior environmental adaptability, directional control, and task responsiveness. For example, magnetic fields enable remote, high-precision navigation, while photothermal or enzyme-driven elements provide localized actuation and on-demand functional activation; their combination thus affords both navigational control and therapeutic targeting. Moreover, hybrid systems can dynamically switch between propulsion modes to accommodate the varying physicochemical conditions encountered across different tissue regions. In 2020, Yuan et al. developed a Janus micromotor powered by both platinum nanoparticle–catalyzed reactions and Fe_2_O_3_-mediated magnetic rotation [29]. This dual-engine design allowed adaptive behavior to suit different application requirements, and its enhanced locomotion combined with localized fluid flows resulted in a twofold increase in its ability to capture and eradicate Staphylococcus aureus. Overall, combined propulsion epitomizes the trend toward multifunctional, intelligent MNRs and is a critical pathway for advancing their clinical applicability.

## 3 Micro/nanorobots for Bacteria elimination

### 3.1 Antimicrobial agent delivery

**Fig. 1:**
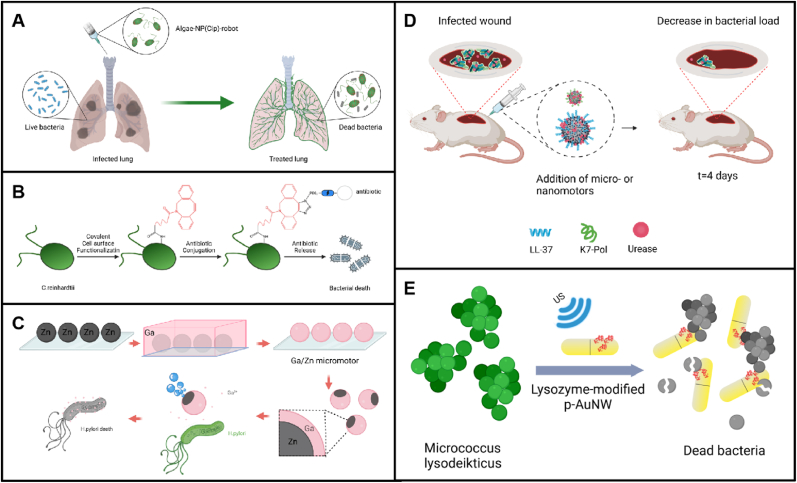
**Antimicrobial agent-based bactericidal strategies of MNRs.** A) Schematic illustration of algae-nanoparticle hybrid microrobots enabling in vivo targeted antibiotic delivery in the treatment of acute bacterial pneumonia. B) Schematic illustration of surface-modified microalgae for the fabrication of biohybrid C. reinhardtii against bacterial infections. C) Schematic of the production and antibacterial mechanism of Janus Ga/Zn microrobots. D) Schematic illustration of the AMP- functionalized urease MNRs and their autonomous propulsion for targeting pathogenic infections. E) Schematic of nanomotors with high-efficiency bactericidal capability, combining the antibacterial activity of lysozyme with rapid movement. Image created with BioRender.com.

The current mainstay of treatment for bacterial infections involves antibiotics such as β-lactams, macrolides, aminoglycosides, tetracyclines, and chloramphenicol. These antibiotics disrupt bacterial cell membranes, inhibit deoxyribonucleic acid (DNA) replication and repair, hinder protein synthesis, and interfere with essential metabolic processes [30,31]. However, the overuse of antibiotics can lead to bacterial resistance. In contrast, utilizing micro-/nanorobots as carriers for antibiotic delivery can enhance the concentration of effective antibiotics, decrease the required dosage, and reduce the probability of resistance development. Zhang et al. designed a microrobot for delivering antibiotics in vivo to treat acute bacterial pneumonia. (Fig. 1A) [32]. The microrobot was composed of *Chlamydomonas reinhardtii* microalgae functionalized with neutrophil membrane-coated and polymeric nanoparticles (NPs) loaded with antibiotic (denoted ‘algae-NP-robot’). In the mouse model, treatment with algae-NP-robots loaded with ciprofloxacin (CIP) resulted in a 3 order of magnitude reduction in bacterial load caused by *Pseudomonas aeruginosa* compared to the negative control, and a 1 order of magnitude reduction compared to static algae-NP(CIP) and NP(CIP). Additionally, the survival rate of the mice was significantly improved. In 2021, Gademann et al. developed biologically driven active microrobots utilizing *Chlamydomonas reinhardtii* as a drug delivery vehicle (Fig. 1B) [33]. By loading antibiotics such as vancomycin and ciprofloxacin onto the surface of *Chlamydomonas reinhardtii*, the antibiotics were transported by the movement of the algae itself. Additionally, the inherent phototropism of the algae was exploited to control the direction of drug transport to the bacterial infection site, where the drugs were released upon ultraviolet (UV) light. This system exhibited potent antimicrobial effects against *Staphylococcus aureus* and *Escherichia coli*, with colony-forming units (CFU) reduced by 3 orders of magnitude compared to the untreated group. A notable drawback is the reduced ability of light to penetrate tissues, which restricts the application of these systems primarily to skin-related infections. Additionally, the potential photodamage to healthy cells further limits their applicability. To address this limitation, Gademann et al. (2023) designed and engineered biohybrid microrobots employing a thiol-mediated self-extinction antibiotic release strategy [34]. Vancomycin was conjugated to a self-extinction linkage platform functionalized on the cell surface. Vancomycin conjugate 7 was produced and chemically bonded to the surface of the green microalga *C. reinhardtii* through a two-step process. Following exposure to a sulfhydryl-reducing agent, the antibiotic dissociated from the biohybrid algal microrobots, exhibiting potent suppression of *Bacillus* and *Staphylococcus* bacterial proliferation.

Additionally, non-antibiotic antibacterial agents can circumvent conventional resistance mechanisms without promoting the development of bacterial antibiotic resistance. Metal-based nanoparticles, owing to their multiple physicochemical modes of action and potent antimicrobial efficacy, have been widely applied in medical devices and consumer products [35]. In 2017, Vilela and colleagues introduced bubble-driven Mg/Ag Janus microrobots that effectively kill *Escherichia coli* [36]. The self-propulsion of these microrobots promoted contact with pathogens, significantly improving their antimicrobial performance. Quantitative analysis via live/dead fluorescence staining revealed that the microrobots achieved >80 % bacterial eradication within 15 min. Recently, Dong and colleagues developed helical graphene microrobots modified with Ag and containing Fe_3_O_4_ nanoparticles, which were actuated by rotating magnetic fields. These microrobots exhibited potent antibacterial activity against *Escherichia coli* [37]. Gallium (Ga), a liquid metal, is naturally biocompatible and degradable in weakly acidic environments, showing significant potential for biomedical applications. Lin et al. designed a Janus microrobot consisting of a zinc (Zn) core and a gallium (Ga) shell (Fig. 1C) [38]. This microrobot was driven by hydrogen bubbles produced via the spontaneous reaction of the Zn core with gastrointestinal acid, a process further intensified by the Ga-Zn galvanic effect. The biocompatible Ga/Zn microrobots were completely degradable in gastrointestinal acid, releasing Ga^3+^ ions that demonstrated potent antibacterial properties against pathogenic bacteria, including *Helicobacter pylori.* While free Ga (4 mg/mL) achieved approximately 99.9 % bactericidal efficiency, Ga/Zn microrobots required only 1 mg/mL to attain comparable efficacy through enhanced targeted delivery and Zn^2+^-mediated synergistic antibacterial action. Besides, metal-organic frameworks (MOFs) can serve as nanocarriers for antibiotic delivery, and the combination of MNRs and MOFs shows great potential for enhancing the efficacy of MNRs in various applications. Liu et al. reported a series of self-powered MOF microrobots [39], which utilized the spontaneous degradation of MOF microrobots in water to release ion components that constituted the robots. These ions not only served as fuel for efficient self-propulsion through ion diffusion-electrophoresis, but also acted as antimicrobial agents to kill *Escherichia coli*, significantly accelerating wound healing in an in vivo bacterial infection wound model.

Although metal ions exhibit remarkable antimicrobial activity, metal-based micro-nanorobots are often associated with high cytotoxicity; by contrast, antimicrobial peptides (AMPs), with their high biological specificity and excellent environmental friendliness, represent a more ideal alternative [[40], [41], [42]]. Arqué et al. used urease to deliver AMPs onto silica-based micro- and nanoparticles, achieving targeted therapy by directing them to the site of infection(Fig. 1D). ^43^Two of the AMPs, LL-37 and K7-Pol, were employed. LL-37 exhibits antibacterial activity, enhances wound healing, and regulates immune responses, while K7-Pol, derived from wasp venom, demonstrates broad-spectrum activity. Although these peptides exhibit potent antimicrobial properties, they are vulnerable to protease degradation. In the mouse model, the researchers successfully delivered AMPs using MNRs, with LL-37-urease micromotors and K7-Pol-urease nanomotors significantly reducing bacterial load by 2 and 3 orders of magnitude, respectively, reaching levels that can be cleared by immune response.

AMPs are frequently employed to inactivate bacteria. However, their use can also contribute to the development of resistance in microorganisms. Another natural antimicrobial agent has attracted attention due to its biocompatibility and reduced potential to induce antimicrobial resistance. Lysozyme is an enzyme that hydrolyzes 1,4-β-linkages between N-acetylmuramic acid and N-acetyl-d-glucosamine residues in peptidoglycan from the cell wall, leading to cell wall degradation and subsequent bacterial death [44]. Notably, the application of free lysozyme for bacterial eradication is frequently hindered by its low stability and limited reusability. Consequently, diverse solid supports, such as nanoparticles, have been integrated to enhance its stability and reusability [45]. Wang and his team were the first to report lysozyme-modified Au nanowires (AuNWs) (Fig. 1E) [46]. Ultrasonic propulsion enabled these nanorobots to move rapidly, increasing the contact between lysozyme and pathogens, preventing the accumulation of dead bacteria on the nanorobot surface, and significantly enhancing their antimicrobial efficacy. The porous structure of the nanorobots provided a larger surface area, enabling them to carry more lysozyme than non-porous gold nanowires, further enhancing antibacterial efficacy. In tests against *Escherichia coli*, these nanorobots achieved over 80 % sterilization within 5 min.

Bacteriophages are viruses capable of infecting and lysing specific bacteria, exhibiting self-replicating and host-specific characteristics. Leveraging these properties, bacteriophages have shown potential as antimicrobial agents, particularly in combating bacterial infections, including multi-drug resistant strains [47]. In 2017, Li et al. first conjugated the polyvalent bacteriophage PEL1 to magnetic colloidal nanoparticle clusters (CNCs), achieving approximately 88.7 % removal of *Escherichia coli* and *Pseudomonas aeruginosa* biofilms, while free phages only removed about 35 % of the biofilm [48]. The researchers suggested that the magnetic properties of the phage–CNC complex enhanced biofilm penetration and physical disruption under a magnetic field, providing valuable insights into the application of bacteriophages in antimicrobial MNRs.

### 3.2 Stimuli-responsive antimicrobial therapy

**Fig. 2:**
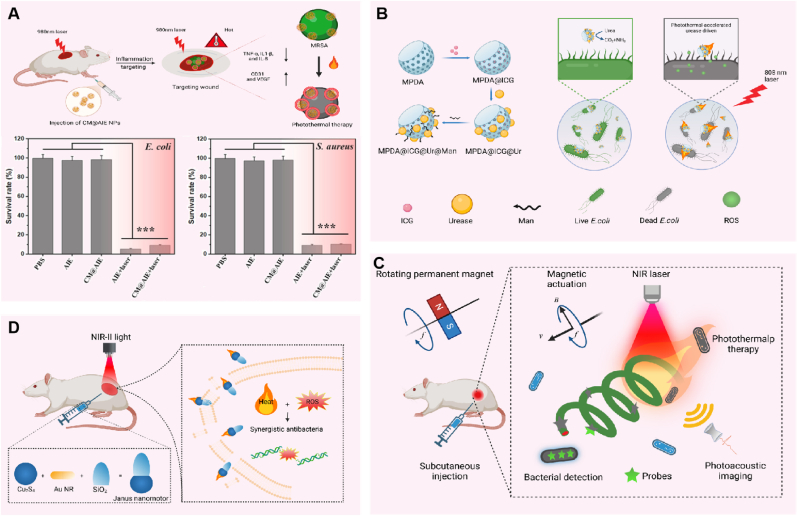
**Phototherapy-based bactericidal strategies of MNRs.** A) Schematic representation of CM@AIE NPs and their antibacterial efficacy in the treatment of skin infections. Reproduced with permission [55]. Copyright 2023, American Chemical Society. B) Synthesis route of MPDA@ICG@Ur@Man nanorobot and photothermal acceleration of the driving and phototherapy processes. C) Schematic of PDA-MSP-based treatment for MDR KP infection. D) Schematic illustration of NIR-II light-driven dual plasmonic (AuNR-SiO_2_-Cu_7_S_4_) antibacterial nanorobots applied in photoacoustic imaging (PAI)-guided synergistic photothermal and photocatalytic therapy. Image created with BioRender.com.

Over the past few years, stimuli-responsive therapies—including light, ultrasound, and microenvironment-triggered approaches—have attracted considerable interest in the antimicrobial field. Among these, photothermal therapy (PTT) is a new type of green therapy that does not rely on antibiotics, and has the excellent characteristics of being controllable, non-invasive, non-resistant, low-toxicity, and low-hemolysis [49]. PTT employs photothermal agents (PTAs) to transform light energy into thermal energy, resulting in irreversible damage to bacteria, leading to protein denaturation and disruption of the membrane structure [50,51]. For instance, emerging PTA aggregation-induced emission (AIE) molecules have demonstrated great promise in therapeutic applications against drug-resistant bacterial infections [[52], [53], [54]]. Nevertheless, challenges such as insufficient targeting of infected lesions and limited penetration into Gram-negative bacterial cell membranes still hinder therapeutic efficacy. To address this limitation, Tang et al. developed bionic neutrophil-like AIE nanorobots (CM@AIE NPs) for targeted localization at inflammatory sites and effective photothermal therapy (Fig. 2A) [55]. By incorporating neutrophil membranes on their surface, CM@AIE NPs can mimic the behavior of source cells and interact with immunomodulatory molecules that would typically target endogenous neutrophils. By combining the absorption of AIE luminophores (AIEgens) in the near-infrared region with outstanding photothermal performance, precise targeting and treatment at the site of inflammation can be realized, reducing harm to adjacent healthy tissues. Additionally, CM@AIE NPs-mediated PTT was activated by in vivo 980 nm laser irradiation, which extended the treatment depth. In vitro, the bactericidal efficiency of the nanorobots against *Staphylococcus aureus* and *Escherichia coli* reached 93.9 % and 94.4 %, respectively. In vivo, CM@AIE NPs combined with PTT reduced the bacterial count at the infection site by 90.6 %, demonstrating a more efficient antibacterial ability compared to conventional methods.

In addition to PTT, photodynamic therapy (PDT) represents another promising light-responsive strategy, utilizing ROS to inactivate bacteria [56,57]. As mobile carriers of photosensitizers, MNRs can enhance the utilization of oxygen molecules and the diffusion of ROS, thereby achieving improved photodynamic therapy. For example, in 2019, Ma et al. developed a urease-driven magnetic hollow porous silica microrobot carrying a highly effective photosensitizer [58]. Under 450 nm light excitation, this microrobot generated ROS and rapidly diffused ^1^O_2_ through a self-propelled mechanism to effectively kill *Escherichia coli*. In 2025, Deng and colleagues developed a NIR-driven nanomotor, termed CSIL, by co-loading indocyanine green (ICG) and lysostaphin onto spiny yolk-shell structured C/SiO_2_@C nanoparticles [59]. Upon NIR irradiation, the CSIL nanomotors were able to penetrate bacterial biofilms, where the synergistic action of ICG-generated ROS and lysostaphin-mediated cell membrane hydrolysis resulted in a bactericidal efficiency of approximately 99.7 %.

However, single treatment modalities have inherent limitations, such as the non-selective and high-intensity laser used in PTT, and the limited diffusion distance (tens to hundreds of nanometers) and short half-life (3.5 μs) of photo-activated singlet oxygen (^1^O_2_) in PDT. The combination of PTT and PDT can destroy the proteins and phospholipids in cell membranes at high temperatures, while also facilitating the penetration of toxic ROS into the bacterial wall, ultimately achieving a "1 + 1 > 2″ antimicrobial effect [[60], [61], [62]]. A light-thermal accelerated urease-driven bowl-shaped mesoporous polydopamine nanorobot (MPDA@ICG@Ur@Man) was reported (Fig. 2B) [63]. By enhancing its motion, the nanorobot increased the contact between antimicrobial agents and bacteria, thereby achieving antibiotic-free synergistic PTT/PDT antimicrobial activity. The bowl-shaped mesoporous polydopamine nanoparticles were synthesized as the core, and indocyanine green (ICG) was loaded onto the nanoparticles via π-π stacking and electrostatic adsorption, providing a platform for photosensitizer delivery. Notably, the light-thermal accelerated bowl-shaped nanorobot enhances urease activity under the local heat generated by PTT, resulting in improved motility. At a single wavelength, the enhanced motion and the synergistic combination of PTT/PDT significantly improved the antibacterial effect. In vitro experimental results demonstrated that the intelligent nanorobot could kill 99 % of *Escherichia coli*.

In addition, light-responsive therapies can be integrated with imaging techniques to enable imaging-guided treatment of bacterial infections. In 2020, Chen and colleagues developed a multifunctional magnetic microswimmer by coating magnetized Spirulina sp. (MSP) with polydopamine (PDA) (Fig. 2C) [64]. The PDA coating enhanced the photoacoustic (PA) signals and photo-thermal effects, thereby allowing the magnetic microswimmer to perform PA-based imaging and photothermal antimicrobial treatment, reducing the survival rate of multidrug-resistant *Klebsiella pneumoniae* (MDR KP) to below 1 % at 400 μg/mL, achieving the integration of therapy and diagnosis. In 2023, Song et al. developed NIR-II light-activated dual-plasmonic (AuNR-SiO_2_-Cu_7_S_4_) antimicrobial nanorobots (Fig. 2D) [65], which were utilized for photoacoustic imaging (PAI)-assisted combined photothermal and photocatalytic treatments for bacterial infections. The photothermal efficiency of these nanorobots was increased by approximately 20 % due to the strong plasmonic coupling and improved energy transfer between the Au nanorods (Au NRs) and Cu_7_S_4_ components. The kinematic behavior of the nanorobots facilitated transdermal penetration and enhanced the interaction between substances and bacteria. Moreover, the directional navigation and synergistic antimicrobial effects of the nanorobots were effectively controlled by light synchronization. By combining active motility with enhanced antimicrobial activity, the system achieved up to 97.8 % antimicrobial efficiency in a mouse model of abscess infection. However, it is important to note that although phototherapy-based MNRs bactericidal strategies have shown great potential, the limitation of light penetration remains a significant clinical challenge [66]. Insufficient light penetration may lead to poor local therapeutic outcomes, and compensating for this limitation with high light intensity may cause thermal damage to normal tissues and photo-toxic reactions, further increasing the risk of complications in patients. Additionally, the degradation products of photothermal nanocomposites may accumulate in the body and lead to acute/chronic tissue toxicity [67]. Therefore, more experiments and observations are needed to assess their long-term biological safety.

Sonodynamic therapy (SDT), activated by ultrasound, possesses excellent tissue penetration capabilities and shows unique potential for treating deep infections [68]. For example, Wu et al. developed a multifunctional platform for acoustic kinetic therapy to treat osteomyelitis caused by MRSA infection [69]. The system was composed of a single-atom doped porphyrin metal-organic framework (HNTM-Pt@Au), driven by Au nanorods (Au NRs). Electron transfer efficiency and oxygen adsorption were significantly enhanced by Pt single atoms under US excitation, endowing the porphyrin-based acoustic sensitizer with remarkable acoustic kinetic antimicrobial properties. After only 15 min of ultrasound treatment, nearly 99.9 % of MRSA bacteria were eliminated. In a model of osteomyelitis infected by MRSA, the ultrasound (US) + RBC-HNTM-Pt@Au system completely cleared the infection after a 4-week treatment regimen involving effective 30-min sonodynamic therapy (SDT) sessions. In 2025, Song et al. designed a nanorobot by loading the sonosensitizer protoporphyrin IX (PpIX) onto ZIF-8 metal–organic frameworks (MOFs) modified with poly-L-arginine (PArg) [70]. Under ultrasound stimulation, the nanorobot effectively eradicated Streptococcus pneumoniae while simultaneously leveraging immunogenic bacterial debris for in situ vaccination. This approach induced both mucosal immune responses and immune memory, enabling not only rapid bacterial clearance but also long-term prophylactic protection.

In addition to external stimuli like light and ultrasound, internal infection-associated microenvironments have also been leveraged to guide smart therapeutic interventions. Bacterial infection sites typically exhibit distinctive characteristics such as low pH, high hydrogen peroxide (H_2_O_2_) levels, and overexpression of enzymes and toxins. These features enable the infected area to serve as a trigger signal for micro/nanomotors, promoting targeted drug release at precise locations. This approach can enhance drug accumulation at the infection site, reduce systemic distribution, decrease dosing frequency, and effectively slow the development of antimicrobial resistance [71]. For example, Song et al. developed a biodegradable magnesium-based micromotor (Mg-Tob micromotor) loaded with tobramycin for synergistic treatment of sepsis [72]. This micromotor utilized the acidic environment in the peritoneal fluid of septic mice as a trigger signal, generating hydrogen gas through the reaction of magnesium with water to actively deliver the drug. In the high-concentration inflammation and bacterial infection microenvironment, the Mg-Tob micromotor significantly improved the survival rate of septic mice to 87.5 %, compared to approximately 50 % survival with conventional treatment.

## 4 Micro/nanorobots for eradication of bacterial biofilms

Epidemiological data indicate that approximately 80 % of chronic bacterial infections [73], such as osteomyelitis [74], cystic fibrosis-related lung infections [75], dental plaque [76], urinary tract infections [77], and ear infections [77,78], are associated with biofilm formation. The complex architecture and multiple defense mechanisms of antibiotic-resistant bacterial biofilms significantly hinder antibiotic penetration and therapeutic efficacy. Biofilms are primarily composed of extracellular polymeric substances (EPS) secreted by bacteria, including polysaccharides, proteins, lipids, and extracellular DNA (eDNA) [78]. These EPS components provide protective functions, shielding bacteria from antibiotics, metal ions, oxidants, and host immune responses. The high viscosity of EPS and the cross-linking between polymer molecules create a dense matrix that severely impedes the penetration of antibiotics, resulting in poor drug permeability [79]. In addition, the viscous and compact structure of biofilms restricts nutrient diffusion, leading to reduced metabolic activity of the bacteria within. This low metabolic state slows bacterial growth and division, rendering antibiotics—typically more effective against rapidly dividing cells—less effective, thereby conferring intrinsic resistance to the biofilm-residing bacteria [80]. More importantly, within the biofilm matrix, bacteria can exchange antibiotic resistance genes through horizontal gene transfer, facilitating the rapid spread of resistance throughout the bacterial community [81]. Consequently, even bacterial populations initially sensitive to antibiotics may acquire resistance during the course of treatment, complicating infection management.

In summary, the barrier properties of the biofilm matrix result in poor penetration of therapeutic agents. Enhancing drug permeability and reducing EPS-mediated interference are, therefore, key strategies for overcoming biofilm-associated resistance [82]. In recent years, MNRs have emerged as a promising approach for combating biofilms. These devices can penetrate biofilms precisely through targeted delivery and release of therapeutic agents, thereby significantly improving drug diffusion. Furthermore, MNRs can disrupt the biofilm matrix and bacterial cell walls through ROS generation or mechanical force, leading to enhanced antibiofilm efficacy. The synergistic effects of these mechanisms provide new hope for effective treatment strategies.

**Fig. 3:**
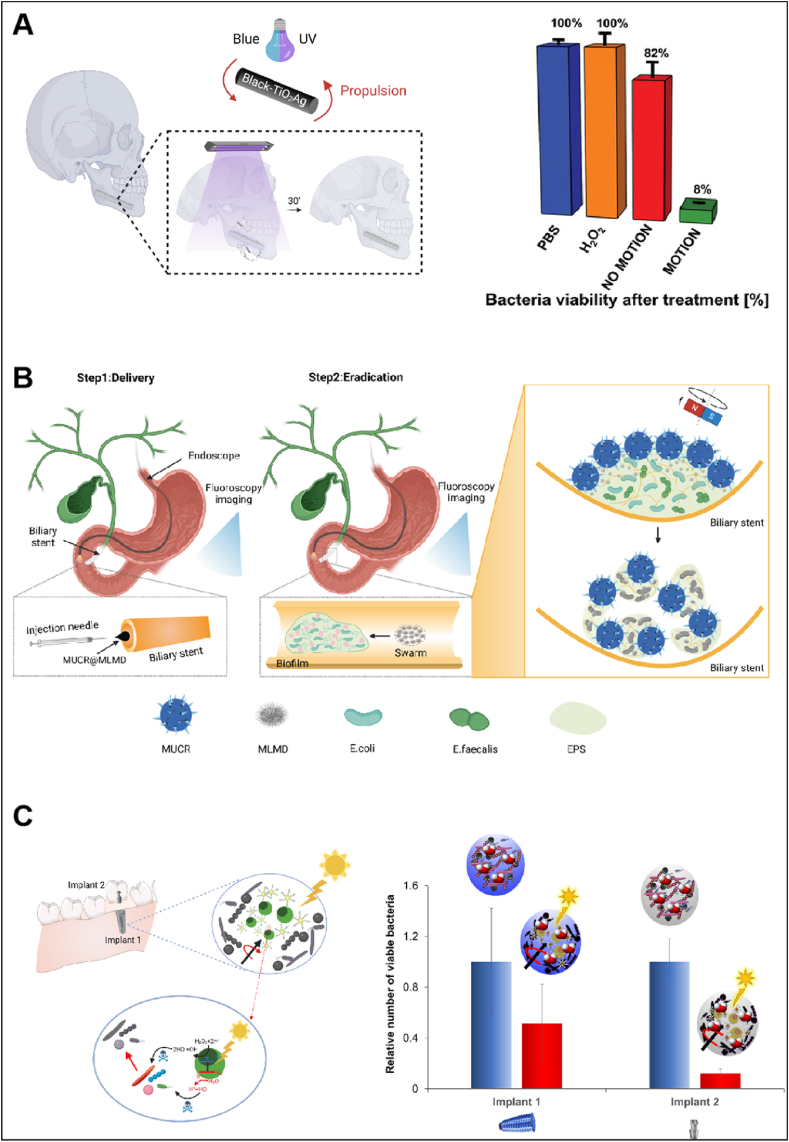
**Antibacterial strategies targeting biofilms.** A) Schematic representation of light-driven B-TiO_2_/Ag nanorobots and their antibacterial efficacy against multispecies biofilm on titanium miniplates. Reproduced with permission [84]. Copyright 2022, Wiley-VCH GmbH. B) Schematic Representation of the removal of biofilm from biliary stents by employing MUCR@MLMDs swarm. C) Schematic representation of photoactive Fe_3_O_4_@PEI/BiVO_4_ magnetic microrobots and their oral antibiofilm efficacy. Reproduced with permission [90]. Copyright 2022, American Chemical Society. Image created with BioRender.com.

ROS are generated by MNRs through catalytic reactions, which play a key role in biofilm removal. For instance, Photocatalysts like titanium dioxide (TiO_2_), when exposed to light, can produce ROS that are highly oxidative, capable of degrading the biofilm's extracellular matrix, interfering with bacterial metabolism, and even killing bacteria. This photocatalytic process effectively disrupts biofilms, making microrobots an important tool in antimicrobial therapy. In 2021, a hybrid enzyme/photocatalytic microrobot based on TiO_2_/CdS nanotube bundles immobilized with urease was developed by the Pumera group [83]. These microrobots were mobile when exposed to urea, with TiO_2_/CdS nanotube bundles acting as catalysts to produce ROS that killed bacteria. Nearly 90 % of the bacterial biofilm was removed by these microrobots after 2 h of light exposure. In 2022, the same group introduced a tubular black TiO_2_/Ag (B-TiO_2_/Ag) nanorobot for the treatment of oral biofilm(Fig. 3A) [84]. These nanorobots were capable of absorbing a wide range of light, from UV to visible light, which controlled their multimodal movement. The rapid rotation and random autonomous motion of B-TiO_2_/Ag nanorobots made them highly biocompatible, exhibiting enhanced antimicrobial properties through the increased release of ROS and silver ions when exposed to light. Experiments demonstrated that biofilm removal was more effective in the presence of 0.1 % H_2_O_2_ under UV irradiation, resulting in a reduction of bacterial biofilm mass by as much as 92 %. It is important to note that prolonged exposure to blue and UV light is harmful, and that UV and visible light cannot penetrate deeply into muscle tissue.

Mechanical cell lysis serves as a straightforward and efficient approach for disrupting the EPS within biofilms. MNRs generate friction and shear forces during the morphological transformation process, effectively disrupting the EPS and removing the biofilm. However, individual MNRs may have limited motility and disruption efficacy, particularly with large-scale biofilms. The introduction of magnetic drive technology provides a solution. MNRs under an external magnetic field are accurately positioned and produce stronger forces through cluster movement. These robots' synergy and positioning are enabled by magnetic fields, allowing them to destroy biofilms in hard-to-reach areas [[85], [86], [87]]. For instance, Sun et al. designed a capsule robot resembling a sea urchin (MUCR), equipped with magnetic liquid metal droplets (MLMDs) as antibacterial agents, fabricated from natural sunflower pollen (Fig. 3B) [88]. The effectiveness of MUCR@MLMDs swarm in eliminating a complex bacterial biofilm mixture inside biliary stents obtained from patients was investigated. Microswarm formation was triggered by an external magnetic field, inducing the MLMDs to change shape into rods and spheroids featuring sharp edges. The natural microspines of the MUCRs, combined with the sharp structures of the MLMDs, mechanically disrupted the compact biomatrix and numerous encapsulated bacterial cells, thereby resulting in synergistic biofilm eradication. Additionally, the movement of the swarm can significantly improve the biofilm removal efficiency. The MUCR@MLMD group exhibited a bactericidal efficiency of over 99 % in killing bacterial cells in biofilm, which was considerably higher than that of the static MUCR@MLMD.

Single antimicrobial methods often encounter issues such as drug resistance and therapeutic limitations. To address these challenges, synergistic strategies that combine ROS systems with physical disruption methods are particularly important. ROS can effectively kill bacteria by inducing oxidative stress, while magnetic MNRs can disrupt the EPS of biofilms using mechanical forces. The combined action of these two approaches can effectively overcome the difficulties posed by complex biofilms. In 2019, catalytic antimicrobial robots (CARs) were developed by H. Koo and colleagues for the precise, controlled killing, degradation, and removal of biofilms [89]. CARs utilized iron oxide nanoparticles (NPs) that possess dual catalytic-magnetic functionalities. These nanoparticles generated bactericidal radicals that helped decompose the EPS matrix of biofilms, which were then mechanically disrupted and physically removed by forces generated by a magnetic field. Two distinct CARs were created: the biohybrid CAR and the 3D molded CAR. The biohybrid CAR was made up of nanoparticles and biofilm fragments that were assembled into a superstructure. When activated by an external magnetic field, this superstructure disrupted and completely removed the biofilm, preventing its regeneration. The 3D molded CAR, on the other hand, was a polymer-based soft robot that incorporates catalytic-magnetic NPs, molded into custom 3D printed shapes to perform specific tasks within enclosed spaces. For instance, the Vane-shaped CAR was designed to remove biofilm from curved walls, while the helicoid-shaped CAR can drill through biofilm plugs. In another study, a hybrid motor (Fe_3_O_4_@PEI/BiVO_4_) was developed, composed of highly efficient photoactive BiVO_4_ microparticles and Fe_3_O_4_ nanoparticles, which were assembled via micellar polyethyleneimine (PEI) polymers(Fig. 3C) [90]. The Fe_3_O_4_ material, activated by a transversely rotating magnetic field, was used to facilitate the uniform distribution of oxide species generated by BiVO_4_. Under the rotating magnetic field, the strong force generated by the swarming Fe_3_O_4_@PEI/BiVO_4_ magnetic microrobots effectively disrupted oral biofilms through mechanical means. The combination of magnetic motion and the antimicrobial activity of BiVO_4_-generated oxide species resulted in approximately 50 % and 90 % biofilm removal on two types of dental implants, respectively.

The above studies demonstrate the effectiveness of the synergistic strategy of the ROS system and physical disruption in biofilm removal, further demonstrating the potential of MNRs in antimicrobial therapy. However, it is important to note that reactive oxygen generating systems can be rapidly and effectively cytotoxic to bacteria, but the majority of them damage healthy cells and rely on an aerobic condition. Magnetically driven robots can generate mechanical forces to disrupt biofilm EPS, but most of these magnetic materials are not biodegradable or biocompatible.

## 5 Discussion

### 5.1 Biocompatibility and biodegradability

The overall safety and biocompatibility of antimicrobial MNRs primarily depend on their propulsion mechanisms and fabrication materials [91]. Currently, the propulsion strategies of MNRs can be broadly categorized into two types: those powered by chemical reactions and those driven by external energy fields without the need for chemical fuels. For chemically propelled MNRs, research efforts have mainly focused on reducing the reliance on toxic hydrogen peroxide, instead utilizing more biocompatible alternative fuels such as acids, water, or substrates specific to certain enzymes. In particular, enzyme-powered MNRs have attracted significant attention due to their ability to achieve in situ fuel generation within biological environments. However, their propulsion mechanisms remain to be fully elucidated. On the other hand, fuel-free propulsion methods, such as NIR light and ultrasound, offer excellent tissue penetration and non-invasive characteristics, although the potential for tissue damage under high-intensity energy exposure warrants careful consideration. Future studies should further explore the integration of current propulsion strategies with advanced manipulation techniques, such as optical tweezers, magnetic resonance imaging (MRI), and holography, to enhance the controllability and adaptability of MNRs within biological environments.

**Fig. 4:**
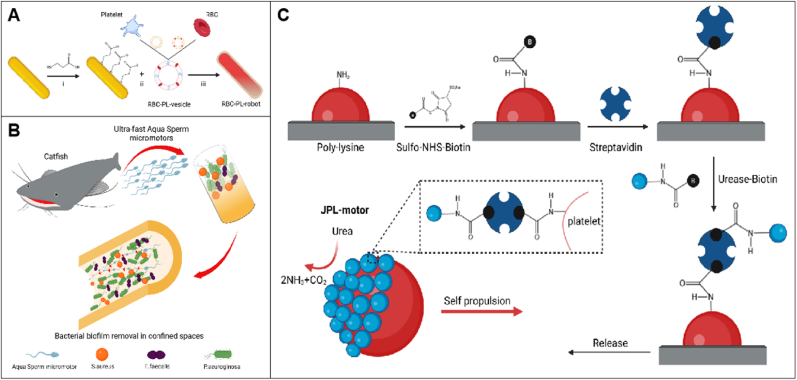
**Biocompatible micro/nanorobots** A) Fabrication of RBC-PL-robots: (i) MPA was utilized to functionalize the gold surface of the Au NW robots; (ii) Hybrid membranes, created by fusing RBC and PL membranes at a 1:1 protein weight ratio, were then applied to coat the modified nanorobots; (iii) The RBC-PL-robots were prepared following a 5-min sonication step. B) Schematic of ultra‐fast speed Aqua Sperm microrobots derived from North African catfish. C) Schematic of the fabrication of JPL-motors. Platelets were attached to PLL and functionalized with urease using a biotin-streptavidin-biotin linkage. Image created with BioRender.com. (For interpretation of the references to colour in this figure legend, the reader is referred to the Web version of this article.)

On the other hand, the biocompatibility of fabrication materials is equally critical. Interactions between MNRs and various biological tissues in vivo may lead to adverse effects, such as inflammation and fever. Additionally, as foreign invaders, MNRs for in vivo drug delivery are prone to triggering passive immune clearance, resulting in the possibility of being engulfed by phagocytes before accomplishing their targets or increasing the retention effect through bioadhesion and the reticuloendothelial system, which reduces the efficacy. Surface camouflage coatings represent the most widely used strategy for immune evasion. Classic biocompatible materials, such as polymers and silica dioxide, are commonly incorporated onto the surfaces of MNRs via physical or chemical methods to suppress immune activation. Moreover, cloaking antimicrobial MNRs with cell membranes derived from various cell types has emerged as a promising avenue in the field. This strategy effectively prevents immune attacks and biofouling, thereby reducing the risk of functional failure and prolonging the operational lifespan of MNRs [92,93]. Ultrasonic nanomotors were developed by Wu and colleagues through the fusion of Au nanowire robots with red blood cell (RBC) nanobubbles [94]. Subsequently, magnetic helical nanorobots made of Ni/Au/Pd and coated with human platelet (PL) membranes were created [95]. Ultrasonic Au nanowire robots with hybrid membranes from RBCs and PLs were later constructed(Fig. 4A) [96]. Rapid, effective, and long-lasting ultrasonic drive in whole blood was exhibited by these biohybrid nanorobots without significant biological contamination. Furthermore, Its adsorption capacity against methicillin-resistant *Staphylococcus aureus* (MRSA) was 3.5 times higher than that under static conditions, and its neutralizing efficiency against α-toxin was also significantly improved, with the hemolysis rate decreasing from 40 % to 5.5 %, indicating that the robot was capable of efficiently removing both pathogens and toxins simultaneously, demonstrating strong antibacterial and detoxification capabilities.

Cell membrane camouflage technology significantly enhances the immune evasion ability of micro/nanobots, but challenges such as the source of the membrane and loading efficiency still exist. Membrane sources are mainly limited to red blood cells, platelets, and cancer cells. How to diversify membrane sources to improve the targeting ability and therapeutic effect of nanobots in specific pathological conditions remains an unresolved issue. The composition and structure differences of various cell membranes directly affect the loading efficiency of nanoparticles. For example, cholesterol-rich membranes may have a higher adsorption affinity for certain types of nanoparticles, while membrane fluidity may influence the transmembrane efficiency of nanomaterials. Additionally, factors such as surface charge, membrane protein types, and membrane flexibility also impact the binding mode and efficiency of nanomaterials. Therefore, finding suitable membrane sources and optimizing loading methods is key to improving the performance of nanobots. To address these challenges, Common loading methods include extrusion, sonication, and electroporation. Extrusion is stable but not scalable, while sonication requires optimization to reduce drug leakage and protein denaturation. Electroporation maintains membrane integrity and reduces protein loss, but its impact on cell damage and viability needs further study [[88], [89], [90]].

Another strategy involves fabricating antimicrobial MNRs by combining them with various cell types through biohybridization, such as non-motile pollen [97], spores [98], microalgae [99], and motile cells with flagella, including spermatozoa [100,101] and bacteria [102,103]. Catfish aqua sperm cells were utilized by Pumera et al. to disrupt biofilms of *Pseudomonas aeruginosa*, *Staphylococcus aureus*, and *Enterococcus faecalis*(Fig. 4B) [104], achieving a reduction of approximately 87 % in bacterial load within the biofilm in just 1 min. However, the biocompatibility of such cells remains a concern, as introducing non-human cells may cause allergic reactions or increase the pathogenicity of specific bacterial strains. Therefore, preclinical experiments are required to assess these potential risks and ensure both safety and efficacy. Utilizing humanized cells or biocompatible coatings offers a more favorable alternative. For instance, in 2020, Wang's group developed a urease-powered Janus platelet micromotor (JPL-motors) by asymmetrically immobilizing urease on natural platelets(Fig. 4C) [105]. These micromotors exhibited self-propelled and sustained motion in urea environments, facilitated by enzymatic fuel breakdown without requiring a motor drive. Simultaneously, JPL-motors retained the inherent biological functions of PL, enabling efficient targeting of bacteria. After 5 min of incubation in 100 mM urea, the binding efficiency of JPL-motors to *Escherichia coli* was approximately 2 times higher than that of passive PLs. This integration of natural enzymes with PL produced a fully biocompatible micromotor. It is worth noting that bioorthogonal chemistry is an important tool in biological hybridization techniques, enabling the efficient coupling between biological and non-biological materials. For example, through bioorthogonal reactions such as click chemistry, specific chemical groups can be introduced onto the surface of biological materials, which then react with complementary groups on non-biological materials, facilitating their coupling. Feng et al. developed a photothermal agent, IR780-DBCO, by introducing 3-azido-D-alanine through metabolic labeling, enabling selective labeling of the cell walls of Gram-positive bacteria [106]. Under near-infrared light exposure, these labeled bacteria can be efficiently eradicated, with minimal impact on surrounding normal cells.

These MNRs need to be broken down (e.g., by enzymatic digestion or hydrolysis) after completing their tasks in vivo, with the degradation byproducts being harmless to healthy cells. However, existing research has primarily concentrated on proof-of-concept biodegradable MNRs that lack sufficient in vivo testing. Greater emphasis should be placed on the application of biodegradable materials to enhance their in vivo testing and facilitate clinical translation. The adoption of FDA-approved drug delivery materials, supported by extensive clinical research, will expedite the translation process. Natural polymers, including chitosan, hyaluronic acid, phospholipids, and collagen, have found extensive application in drug encapsulation and delivery systems owing to their superior biocompatibility and controlled degradation properties [107,108]. Nonetheless, designing biodegradable materials capable of performing complex tasks remains challenging, particularly concerning multifunctionality, structural stability, and stimulus responsiveness. Thus, beyond natural polymers, synthetic polymers such as poly (lactic acid)-co-glycolic acid (PLGA), polycaprolactone (PCL), and poly (sebacic acid glycerol ester) (PGS) hold promise for clinical translation into MNRs due to their superior mechanical properties, biodegradability, and immuno-inertness [[109], [110], [111]].

### 5.2 Precise control and visualization

Precise control is of great significance in the antibacterial applications of MNRs. By accurately regulating the robot's motion trajectory, positioning, and drug release, the targeted therapeutic effect can be significantly enhanced while minimizing damage to healthy tissues. The key to achieving this goal lies in selecting nanomaterials suitable for precise control. For example, superparamagnetic iron oxide nanoparticles (SPIO NPs) exhibit excellent performance in responding to external magnetic fields. They can rapidly reach magnetic saturation under the guidance of a magnetic field and be precisely targeted to the desired area. After the removal of the magnetic field, they lose their magnetism, making them ideal materials for achieving precise in vivo targeted delivery [112]. However, precise control still faces several challenges in practical applications. The complex biological environment within the human body, such as blood flow velocity, tissue density differences, and other factors, may affect the precise localization of the robots. Additionally, many micro-nano robots rely on mechanisms responsive to external stimuli (such as magnetic fields, temperature, etc.) for drug release control. Current technologies have not yet fully resolved the issue of synchronized regulation of multiple stimuli [113].

Combining MNRs with visualization techniques is critical for enabling accurate control and real-time tracking, a necessary direction in clinical applications. Various visualization modalities have recently been employed for MNRs imaging, such as magnetic resonance imaging (MRI) [114,115], ultrasound (US) imaging [116,117], fluorescence imaging [118,119], and computed tomography (CT) [120,121]. Nevertheless, each imaging modality presents unique advantages and limitations, with no single technique capable of addressing all challenges in medical MNRs. X-rays offer high resolution and deep penetration but are expensive and pose potential ionization risks. Optical imaging methods are safe and provide high resolution but are restricted by limited tissue penetration. MRI enables deep tissue imaging without requiring contrast agents but necessitates strong magnetic fields and costly infrastructure. US imaging, while limited in spatial resolution, enables real-time monitoring, offers deep tissue penetration, and is both cost-effective and safe. Multimodal imaging integrates the strengths of various techniques and mitigates their limitations by employing multiple physical principles. For instance, PAI combines optical and ultrasound imaging to produce images with high contrast (resulting from light absorption) and high spatial-temporal resolution [[122], [123], [124]]. Similarly, magneto-motor ultrasound imaging (MMUS) leverages ultrasound signals generated by minute motions induced by magnetic fields, achieving high-contrast, high-resolution real-time tracking of magnetic microrobots without radiation exposure and ensuring operational safety [125,126]. Future research may also explore swarming control as a means of reducing technical demands for MNR imaging. This approach can diminish the reliance on high spatial resolution, enhance soft tissue contrast, and facilitate robust in vivo localization [127]. Moreover, swarming control can improve the contrast of specific imaging techniques and enable the incorporation of higher doses of contrast agents within the swarm.

### 5.3 Clinical translation

Antimicrobial MNRs have demonstrated significant success in in vitro experiments; however, their transition from laboratory research to clinical applications remains challenging. Currently, preclinical studies and clinical trials of antimicrobial MNRs remain in their infancy. Although some antimicrobial robots have undergone in vivo testing [[128], [129], [130]], the animal models used have predominantly been small mammals, such as mice. Nonetheless, micro/nanorobot-based therapies must ultimately be validated in large mammals and human subjects. The study by Bladin et al. represents the only current example of clinical application of antimicrobial MNRs [131], underscoring both the potential of MNRs and the pressing need for further research and development.

A key challenge in clinical translation is ensuring the long-term stability of antimicrobial MNRs in vivo. While MNRs exhibit promising biocompatibility in vitro, in the human body, the immune system recognizes these robots as foreign entities, triggering immune responses that lead to their clearance. Additionally, non-biodegradable robots may persist over time, potentially causing tissue damage. We have discussed this issue in detail in the biocompatibility and biodegradability section. Furthermore, MNRs may undergo aggregation, dissolution, or structural damage in complex physiological environments due to variations in temperature, pH, ion strength, etc. For example, metal-based nanomaterials tend to aggregate in high ion strength environments [132], which can affect their interaction with bacteria. Some Fenton reaction-based antimicrobial MNRs may also have their chemical reactions interfered with by the antioxidant system in the body, reducing their antimicrobial efficacy. Additionally, the sustainability of propulsion systems and power sources poses challenges. For instance, robots powered by hydrogen peroxide may experience reduced propulsion capabilities in vivo due to insufficient fuel, while the penetration depth and transmission efficiency of external energy in the body are limited, affecting their long-term mobility. Ideally, antimicrobial MNRs should utilize harmful components present in the infected microenvironment as driving substrates, with the reaction products being either beneficial or harmless to the human body [133].

Moreover, challenges related to the production and cost of MNRs must not be overlooked. Although 3D printing and self-assembly technologies offer innovative approaches to producing MNRs, these methods face significant challenges, including high costs, low efficiency, and stringent requirements for size control and surface modification, all of which exacerbate production difficulties [134]. For example, most 3D printing materials do not possess antimicrobial properties. To confer antimicrobial capabilities to MNRs, specific antimicrobial agents or nanomaterials must be deposited on the body surface of the 3D-printed MNRs or incorporated into their matrix during or after printing. This clearly increases production costs and may also affect production efficiency and material stability. Formulating antimicrobial inks for one-step 3D printing of antimicrobial MNRs could become an effective strategy [135].

### 5.4 Novel strategies to combat bacterial resistance: applications of nanotechnology

The widespread dissemination of bacterial resistance, particularly multidrug-resistant (MDR) strains, has emerged as a major challenge in global public health. The declining efficacy of conventional antibiotic therapies has driven researchers to explore alternative antimicrobial strategies. In recent years, nanomaterials, owing to their unique physicochemical properties, have offered new avenues for overcoming bacterial resistance.

Nanomaterials exert antibacterial effects through diverse mechanisms distinct from the metabolic targets exploited by traditional antibiotics. For example, silver-zinc oxide nanocomposites can release silver (Ag^+^) and zinc (Zn^2+^) ions to generate ROS, leading to disruption of bacterial membrane structures and DNA damage, thereby exhibiting potent antibacterial activity against resistant *Escherichia coli* [136]. In addition, many nanoparticles can directly disrupt bacterial cell walls or membranes through physical mechanisms, which are effective not only against actively proliferating bacteria but also against dormant persister cells, thus bypassing conventional resistance mechanisms [137]. Beyond direct bactericidal effects, nanomaterials also demonstrate significant advantages as drug delivery platforms. By encapsulating or loading antibiotics into nanoparticles, targeted delivery and controlled release can be achieved, reducing systemic toxicity while enhancing bactericidal efficacy against resistant strains. For instance, ampicillin-functionalized nanoparticles have been shown to successfully kill multiple drug-resistant bacteria, such as *Pseudomonas aeruginosa*, *Enterobacter aerogenes*, and a methicillin-resistant isolate of *Staphylococcus aureus* [138]. Furthermore, surface-engineered nanocarriers, such as polymeric nanoparticles with charge-switching capabilities, can release antibiotics in response to the infection microenvironment, effectively overcoming efflux pump-mediated resistance [139]. Bacterial biofilms, which serve as critical survival barriers for resistant bacteria, have also become a key target for nanotechnology-based interventions. Magnetic nanoparticles (MNPs) can be actively driven by external magnetic fields to penetrate and disrupt the biofilm matrix while simultaneously releasing antimicrobial ions to enhance therapeutic efficacy. For example, the poly(oxanorborneneimide)-based cationic polymeric nanoparticles demonstrated strong biofilm penetration and bacterial membrane-disrupting effects, effectively eliminating the MDR biofilms of *Pseudomonas aeruginosa*, *E. cloacae* complex, and MRSA [140]. In addition, some nanoparticle-based platforms can interfere with bacterial quorum sensing systems, effectively inhibiting biofilm formation and consequently weakening bacterial resistance [141]. Currently, several nanotechnology-based antimicrobial formulations have entered clinical trials, such as liposomal amikacin (Arikace) and liposomal ciprofloxacin (Pulmaquin), providing valuable foundational data for future applications of nanotechnology in antibacterial therapy [142].

In conclusion, nanotechnology effectively overcomes the limitations of conventional antibiotic therapies through multiple mechanisms, offering innovative solutions to address infections caused by resistant bacteria. Considering that MNRs represent a highly integrated and controllable new nanoplatform with unique advantages, such as active penetration of biofilms, precise delivery of antimicrobial agents, and responsiveness to infection microenvironments, we believe that biotechnology based on MNRs will demonstrate even greater potential and application prospects in combating bacterial resistance.

## 6 Conclusion

Antimicrobial MNRs have made significant progress in the removal of bacteria and the eradication of biofilms. In recent years, the surface modification and actuation of nanorobots have been optimized to enable precise targeting of infected areas and effective penetration of biofilms. Nanorobots are capable of physically disrupting the biofilm structure of bacteria, using mechanisms such as magnetic fields or light, thereby enhancing the penetration of antimicrobial drugs [143]. In addition, antimicrobial molecules or drugs are often attached to the surfaces of nanorobots, enabling the targeted delivery and controlled release of therapeutic agents [144]. This combined physical and chemical intervention strategy not only enhances the eradication of drug-resistant bacteria but also effectively mitigates the barrier posed by biofilms to drugs, thereby demonstrating significant potential for treating drug-resistant infections.

The clinical translation of antimicrobial MNRs faces challenges such as long-term safety, production cost, and precision in targeted therapy. Nevertheless, continuous advances in nanotechnology and biomedicine are clarifying the prospects of these robots in antimicrobial treatment. Future research should prioritize improving biocompatibility, degradability, and multifunctionality while advancing their in vivo safety assessment and clinical validation. Advances in manufacturing technology are expected to improve production efficiency and reduce costs, thereby accelerating the clinical application of MNRs.

## CRediT authorship contribution statement

**Xutong Chen:** Writing – review & editing, Writing – original draft. **Yong Li:** Writing – review & editing, Writing – original draft. **Chunhua Wang:** Visualization, Investigation. **Zhiqiang Chen:** Writing – review & editing. **Zhijie Xu:** Writing – review & editing, Supervision, Funding acquisition, Conceptualization. **Fada Xia:** Writing – review & editing, Visualization. **Yuanliang Yan:** Writing – review & editing, Supervision. **Ming Gao:** Writing – review & editing, Supervision.

## Ethics approval and consent to participate

Not applicable.

## Consent for publication

Not applicable.

## Availability of data and materials

Not applicable.

## Funding

This study was supported by grants from the 10.13039/501100001809National Natural Science Foundation of China (82473299), the 10.13039/501100004735Natural Science Foundation of Hunan Province (2024JJ5560), and Hunan Provincial Clinical Medical Research Center for Pediatric Solid Tumors (2023SK4058).

## Declaration of competing interest

The authors declare that they have no known competing financial interests or personal relationships that could have appeared to influence the work reported in this paper.

## Figures and Tables

**Table:** 

	Article list	Copyright	License Number
**1**	Wang, W., Gao, Y., Zhang, M., Li, Y. & Tang, B. Z. Neutrophil-like Biomimic AIE Nanoparticles with High-Efficiency Inflammatory Cytokine Targeting Enable Precise Photothermal Therapy and Alleviation of Inflammation. ACS Nano 17, 7394–7405 (2023).	ACS Publications	License Granted
**2**	Ussia, M. et al. Light-Propelled Nanorobots for Facial Titanium Implants Biofilms Removal. Small 18, 2200708 (2022).	John Wiley and Sons	6030670893584
**3**	Mayorga-Martinez, C. C. et al. Swarming Magnetic Photoactive Microrobots for Dental Implant Biofilm Eradication. ACS Nano 16, 8694–8703 (2022).	ACS Publications	License Granted

## Data Availability

No data was used for the research described in the article.

## References

[1] Huemer M., Mairpady Shambat S., Brugger S.D., Zinkernagel A.S. (2020). Antibiotic resistance and persistence—implications for human health and treatment perspectives. EMBO Rep..

[2] Baran A., Kwiatkowska A., Potocki L. (2023). Antibiotics and bacterial resistance—a short story of an endless arms race. Int. J. Mol. Sci..

[3] Chan Y.L., Chee C.F., Tang S.N., Tay S.T. (2024). Unveilling genetic profiles and correlations of biofilm-associated genes, quorum sensing, and antibiotic resistance in Staphylococcus aureus isolated from a Malaysian Teaching Hospital. Eur. J. Med. Res..

[4] Ge M., Jiang F., Lin H. (2024). Nanocatalytic medicine enabled next-generation therapeutics for bacterial infections. Mater. Today Bio.

[5] Rosini R., Nicchi S., Pizza M., Rappuoli R. (2020). Vaccines against antimicrobial resistance. Front. Immunol..

[6] Collaborators A.R. (2022). Global burden of bacterial antimicrobial resistance in 2019: a systematic analysis. Lancet Lond. Engl..

[7] Yu Y.-L. (2023). Elimination of methicillin-resistant Staphylococcus aureus biofilms on titanium implants via photothermally-triggered nitric oxide and immunotherapy for enhanced osseointegration. Mil. Med. Res..

[8] Al-Khafaji N.S.K. (2024). Prevalence of plasmid-mediated quinolone resistance genes and biofilm formation in different species of quinolone-resistant clinical Shigella isolates: a cross-sectional study. Eur. J. Med. Res..

[9] Grande R., Puca V., Muraro R. (2020). Antibiotic resistance and bacterial biofilm. Expert Opin. Ther. Pat..

[10] Rather M.A., Gupta K., Mandal M. (2021). Microbial biofilm: formation, architecture, antibiotic resistance, and control strategies. Braz. J. Microbiol..

[11] Zhao A., Sun J., Liu Y. (2023). Understanding bacterial biofilms: from definition to treatment strategies. Front. Cell. Infect. Microbiol..

[12] Flemming H.-C., Wingender J. (2010). The biofilm matrix. Nat. Rev. Microbiol..

[13] Soto F., Wang J., Ahmed R., Demirci U. (2020). Medical micro/nanorobots in precision medicine. Adv. Sci..

[14] Llacer-Wintle J. (2021). Biodegradable small-scale Swimmers for biomedical applications. Adv. Mater..

[15] Hu, Y., Zeng, G., Wang, Y. & Yang, D. Nanorobots to treat Candida albicans infection. Research 10, 455. 10.34133/research.0455 PMC11324951 39148662

[16] Rashidy Ahmady A. (2025). Micro‐ and nano‐Bots for infection control. Adv. Mater..

[17] Wang W., Luo H., Wang H. (2024). Recent advances in micro/nanomotors for antibacterial applications. J. Mater. Chem. B.

[18] Su H., Li S., Yang G.-Z., Qian K. (2023). Janus micro/nanorobots in biomedical applications. Adv. Healthcare Mater..

[19] Zhong W. (2024). Miniature robots for battling bacterial infection. ACS Nano.

[20] Ávila B. E.-F. de (2017). Micromotor-enabled active drug delivery for in vivo treatment of stomach infection. Nat. Commun..

[21] Batool N. (2021). An antibacterial nanorobotic approach for the specific targeting and removal of multiple drug-resistant Staphylococcus aureus. Small (Weinh.).

[22] Chaurasia A.K. (2016). Coupling of radiofrequency with magnetic nanoparticles treatment as an alternative physical antibacterial strategy against multiple drug resistant bacteria. Sci. Rep..

[23] H J. (2022). Precisely controlled and deeply penetrated micro-nano hybrid multifunctional motors with enhanced antibacterial activity against refractory biofilm infections. J. Hazard. Mater..

[24] Bhuyan T. (2020). Magnetotactic T-budbots to kill-n-clean biofilms. ACS Appl. Mater. Interfaces.

[25] Liu Y. (2019). Multifunctional magnetic copper ferrite nanoparticles as fenton-like reaction and near-infrared photothermal agents for synergetic antibacterial therapy. ACS Appl. Mater. Interfaces.

[26] Villa K. (2019). Visible-light-driven single-component BiVO4 micromotors with the autonomous ability for capturing microorganisms. ACS Nano.

[27] Maric T., Løvind A., Zhang Z., Geng J., Boisen A. (2023). Near‐infrared light‐driven mesoporous SiO2/Au nanomotors for eradication of Pseudomonas aeruginosa biofilm. Adv. Healthcare Mater..

[28] Xu D. (2021). Enzyme-powered liquid metal nanobots endowed with multiple biomedical functions. ACS Nano.

[29] Yuan, K., Jurado-Sánchez, B. & Escarpa, A. Dual‐Propelled Lanbiotic Based Janus Micromotors for Selective Inactivation of Bacterial Biofilms. doi:10.1002/anie.202011617. 10.1002/anie.202011617 33216439

[30] Zhou C., Wang Q., Cao H., Jiang J., Gao L. Nanozybiotics (2024). Advancing antimicrobial strategies through Biomimetic mechanisms. Adv. Mater..

[31] Cook M.A., Wright G.D. (2022). The past, present, and future of antibiotics. Sci. Transl. Med..

[32] Zhang F. (2022). Nanoparticle-modified microrobots for in vivo antibiotic delivery to treat acute bacterial pneumonia. Nat. Mater..

[33] Shchelik I.S., Molino J.V.D., Gademann K. (2021). Biohybrid microswimmers against bacterial infections. Acta Biomater..

[34] Studer T., Morina D., Shchelik I.S., Gademann K. (2023). Biohybrid microswimmers for antibiotic drug delivery based on a thiol-sensitive release platform. Chem. Eur J..

[35] Zhang Z. (2022). Micro-/Nanorobots in antimicrobial applications: recent progress, challenges, and opportunities. Adv. Healthcare Mater..

[36] Vilela D., Stanton M.M., Parmar J., Sánchez S. (2017). Microbots decorated with silver nanoparticles kill bacteria in aqueous Media. ACS Appl. Mater. Interfaces.

[37] Dong Y. (2020). Graphene-based helical micromotors constructed by “Microscale liquid rope-Coil effect” with Microfluidics. ACS Nano.

[38] Lin Z., Gao C., Wang D., He Q. (2021). Bubble-propelled Janus gallium/zinc micromotors for the active treatment of bacterial infections. Angew. Chem. Int. Ed..

[39] Liu X. (2022). Intrinsic properties enabled metal organic framework micromotors for highly efficient self-propulsion and enhanced antibacterial therapy. ACS Nano.

[40] Mba I.E., Nweze E.I. (2022). Antimicrobial peptides therapy: an emerging alternative for treating drug-resistant bacteria. Yale J. Biol. Med..

[41] Bucataru C., Ciobanasu C. (2024). Antimicrobial peptides: opportunities and challenges in overcoming resistance. Microbiol. Res..

[42] Xuan J. (2023). Antimicrobial peptides for combating drug-resistant bacterial infections. Drug Resist. Updates.

[44] Ragland S.A., Criss A.K. (2017). From bacterial killing to immune modulation: recent insights into the functions of lysozyme. PLoS Pathog..

[45] Xiong J. (2022). Cooperative antibacterial enzyme-Ag-polymer nanocomposites. ACS Nano.

[46] Kiristi M. (2015). Lysozyme-based antibacterial nanomotors. ACS Nano.

[47] Yu P. (2019). Bottom-up biofilm eradication using bacteriophage-loaded magnetic nanocomposites: a computational and experimental study. Environ. Sci. Nano.

[48] Li L.-L. (2017). Enhanced biofilm penetration for microbial control by polyvalent phages conjugated with magnetic colloidal nanoparticle clusters (CNCs). Environ. Sci. Nano.

[49] Zhao Y., Wang Y., Wang X., Qi R., Yuan H. (2023). Recent progress of photothermal therapy based on conjugated nanomaterials in combating microbial infections. Nanomaterials (Basel).

[50] Kejík Z. (2024). Cyanine dyes in the mitochondria-targeting photodynamic and photothermal therapy. Commun. Chem..

[51] Wang Y., Meng H.-M., Song G., Li Z., Zhang X.-B. (2020). Conjugated-polymer-based nanomaterials for photothermal therapy. ACS Appl. Polym. Mater..

[52] Xu Y. (2020). NIR-II emissive multifunctional AIEgen with single laser-activated synergistic photodynamic/photothermal therapy of cancers and pathogens. Biomaterials.

[53] Song N. (2020). Nanomaterials with supramolecular assembly based on AIE Luminogens for theranostic applications. Adv. Mater..

[54] Bai H. (2021). AIEgens for microbial detection and antimicrobial therapy. Biomaterials.

[55] Wang W., Gao Y., Zhang M., Li Y., Tang B.Z. (2023). Neutrophil-like Biomimic AIE nanoparticles with high-efficiency inflammatory Cytokine targeting enable precise photothermal therapy and Alleviation of inflammation. ACS Nano.

[56] Aebisher D. (2024). Photodynamic therapy: past, current, and future. Int. J. Mol. Sci..

[57] Mei L., Zhang Y., Wang K., Chen S., Song T. (2024). Nanomaterials at the forefront of antimicrobial therapy by photodynamic and photothermal strategies. Mater. Today Bio.

[58] Xu D. (2019). Enzymatic micromotors as a mobile photosensitizer platform for highly efficient on-Chip targeted Antibacteria photodynamic therapy. Adv. Funct. Mater..

[59] Deng Y. (2025). NIR light-driven nanomotor with cascade photodynamic therapy for MRSA biofilm eradication and diabetic wound healing. Theranostics.

[60] Overchuk M., Weersink R.A., Wilson B.C., Zheng G. (2023). Photodynamic and photothermal therapies: synergy opportunities for nanomedicine. ACS Nano.

[61] Wei G. (2020). Phototherapy-based combination strategies for bacterial infection treatment. Theranostics.

[62] Yan Z., Wang D., Gao Y. (2023). Nanomaterials for the treatment of bacterial infection by photothermal/photodynamic synergism. Front. Bioeng. Biotechnol..

[63] Liu Y. (2024). Thermal-accelerated urease-driven bowl-like polydopamine nanorobot for targeted photothermal/photodynamic antibiotic-free antibacterial therapy. Adv. Healthcare Mater..

[64] Xie L. (2020). Photoacoustic imaging-trackable magnetic microswimmers for pathogenic bacterial infection treatment. ACS Nano.

[65] Liu L. (2023). Drug-free antimicrobial nanomotor for precise treatment of multidrug-resistant bacterial infections. Nano Lett..

[66] Zhang J. (2025). Implantable, flexible biophotonic device for wireless photodynamic therapy of postoperative infection and tumor recurrence. Device.

[67] Huo J. (2021). Emerging photothermal-derived multimodal synergistic therapy in combating bacterial infections. Chem. Soc. Rev..

[68] Xu Q. (2023). Emerging nanosonosensitizers augment sonodynamic-mediated antimicrobial therapies. Mater. Today Bio.

[69] Yu Y. (2021). Single-atom catalysis for efficient sonodynamic therapy of methicillin-resistant staphylococcus aureus-infected osteomyelitis. ACS Nano.

[70] Song T. (2025).

[71] Zhou Q. (2022). Enzyme-triggered smart antimicrobial drug release systems against bacterial infections. J. Controlled Release.

[72] Song Y. (2023). Micromotor-enabled active hydrogen and tobramycin delivery for synergistic sepsis therapy. Adv. Sci. Weinh. Baden-Wurtt. Ger..

[73] Sharma D., Misba L., Khan A.U. (2019). Antibiotics versus biofilm: an emerging battleground in microbial communities. Antimicrob. Resist. Infect. Control.

[74] Masters E.A. (2019). Evolving concepts in bone infection: redefining “biofilm”, “acute vs. chronic osteomyelitis”, “the immune proteome” and “local antibiotic therapy”. Bone Res..

[75] Thorn C.R. (2021). Tobramycin liquid crystal nanoparticles eradicate cystic fibrosis-related Pseudomonas aeruginosa biofilms. Small (Weinh.).

[76] Jakubovics N.S., Goodman S.D., Mashburn-Warren L., Stafford G.P., Cieplik F. (2021). The dental plaque biofilm matrix. Periodontol. 2000.

[77] Zou, Z. et al. E. coli catheter-associated urinary tract infections are associated with distinctive virulence and biofilm gene determinants. JCI Insight 8, e161461. 10.1172/jci.insight.161461 PMC9977300 36512427

[78] Arciola C.R., Campoccia D., Speziale P., Montanaro L., Costerton J.W. (2012). Biofilm formation in Staphylococcus implant infections. A review of molecular mechanisms and implications for biofilm-resistant materials. Biomaterials.

[79] Schilcher K., Horswill A.R. (2020). Staphylococcal biofilm development: structure, regulation, and treatment strategies. Microbiol. Mol. Biol. Rev. MMBR.

[80] Xiu W. (2022). Potentiating hypoxic microenvironment for antibiotic activation by photodynamic therapy to combat bacterial biofilm infections. Nat. Commun..

[81] Michaelis C., Grohmann E. (2023). Horizontal gene transfer of antibiotic resistance genes in biofilms. Antibiotics.

[82] Lv X. (2023). Recent Nanotechnologies to overcome the bacterial biofilm matrix barriers. Small (Weinh.).

[83] Villa K. (2022). Enzyme-photocatalyst Tandem microrobot powered by urea for Escherichia coli biofilm eradication. Small (Weinh.).

[84] Ussia M. (2022). Light-propelled nanorobots for facial titanium implants biofilms removal. Small (Weinh.).

[85] Zhou H., Mayorga-Martinez C.C., Pané S., Zhang L., Pumera M. (2021). Magnetically driven micro and nanorobots. Chem. Rev..

[86] Gardi G., Ceron S., Wang W., Petersen K., Sitti M. (2022). Microrobot collectives with reconfigurable morphologies, behaviors, and functions. Nat. Commun..

[87] Lu L. (2024). Design and control of the magnetically actuated micro/nanorobot swarm toward biomedical applications. Adv. Healthcare Mater..

[88] Sun M. (2022). Magnetic Microswarm and Fluoroscopy-guided platform for biofilm eradication in biliary stents. Adv. Mater..

[89] Hwang G. (2019). Catalytic antimicrobial robots for biofilm eradication. Sci. Robot..

[90] Mayorga-Martinez C.C. (2022). Swarming magnetic photoactive microrobots for dental implant biofilm eradication. ACS Nano.

[91] Wang S. (2019). Biocompatibility of artificial micro/nanomotors for use in biomedicine. Nanoscale.

[92] Zhang F. (2022). Biomembrane‐functionalized micromotors: biocompatible active devices for diverse biomedical applications. Adv. Mater..

[93] Zeng S. (2023). Cell membrane-coated nanomaterials for cancer therapy. Mater. Today Bio.

[94] Wu Z. (2015). Cell-membrane-coated synthetic nanomotors for effective Biodetoxification. Adv. Funct. Mater..

[95] Li J. (2018). Biomimetic platelet-Camouflaged nanorobots for binding and isolation of biological Threats. Adv. Mater..

[96] Esteban-Fernández de Ávila B. (2018). Hybrid biomembrane–functionalized nanorobots for concurrent removal of pathogenic bacteria and toxins. Sci. Robot..

[97] Yu S. (2023). Magnetic-acoustic actuated spinous microrobot for enhanced degradation of organic pollutants. Ultrason. Sonochem..

[98] Zhang Y. (2019). Real-time tracking of fluorescent magnetic spore–based microrobots for remote detection of C. diff toxins. Sci. Adv..

[99] Choi H. (2023). Microalgae‐based biohybrid microrobot for accelerated diabetic wound healing. Small (Weinh.).

[100] Magdanz V. (2017). Spermatozoa as functional components of robotic microswimmers. Adv. Mater..

[101] Magdanz V., Sanchez S., Schmidt O.G. (2013). Development of a sperm-flagella driven micro-bio-robot. Adv. Mater..

[102] Bastos-Arrieta J., Revilla-Guarinos A., Uspal W.E., Simmchen J. (2018). Bacterial biohybrid microswimmers. Front. Robot. AI.

[103] Stanton M.M. (2017). Magnetotactic bacteria powered biohybrids target *E. coli* biofilms. ACS Nano.

[104] Mayorga‐Martinez C.C. (2021). Swarming aqua sperm micromotors for active bacterial biofilms removal in Confined spaces. Adv. Sci..

[105] Tang S. (2020). Enzyme-powered Janus platelet cell robots for active and targeted drug delivery. Sci. Robot..

[106] Feng T. (2022). Selective inactivation of Gram-positive bacteria in vitro and in vivo through metabolic labelling. Sci. China Mater..

[107] Shields IV C.W., Wang L.L.-W., Evans M.A., Mitragotri S. (2020). Materials for immunotherapy. Adv. Mater..

[108] Jiang, Z. et al. Multiple natural polymers in drug and gene delivery systems. Curr. Med. Chem. 31, 1691–1715. 10.2174/0929867330666230316094540 36927424

[109] Lee J.G., Raj R.R., Day N.B., Shields C.W.I. (2023). Microrobots for biomedicine: Unsolved challenges and opportunities for translation. ACS Nano.

[110] Arif, U. et al. Biocompatible polymers and their potential biomedical applications: a review. Curr. Pharm. Des. 25, 3608–3619. 10.2174/1381612825999191011105148 31604409

[111] Chen C.-K. (2020). Biodegradable polymers for gene-delivery applications. Int. J. Nanomed..

[112] Jeon H. (2016). Poly-paclitaxel/cyclodextrin-SPION nano-assembly for magnetically guided drug delivery system. J. Control. Release Off. J. Control. Release Soc..

[113] Liu Y., Li M., Yang F., Gu N. (2017). Magnetic drug delivery systems. Sci. China Mater..

[114] Go, G. et al. Multifunctional microrobot with real-time visualization and magnetic resonance imaging for chemoembolization therapy of liver cancer. Sci. Adv. 8, eabq8545. 10.1126/sciadv.abq8545 PMC9674283 36399561

[115] Yan X. (2017). Multifunctional biohybrid magnetite microrobots for imaging-guided therapy. Sci. Robot..

[116] Yu J. (2019). Active generation and magnetic actuation of microrobotic swarms in bio-fluids. Nat. Commun..

[117] Wang L. (2021). Guiding drug through interrupted bloodstream for Potentiated Thrombolysis by C-shaped magnetic actuation system in vivo. Adv. Mater..

[118] Zhang Y. (2019). Real-time tracking of fluorescent magnetic spore–based microrobots for remote detection of C. diff toxins. Sci. Adv..

[119] Servant A., Qiu F., Mazza M., Kostarelos K., Nelson B.J. (2015). Controlled in vivo swimming of a swarm of bacteria-like microrobotic flagella. Adv. Mater..

[120] Xu Z. (2019). X-ray-Powered micromotors. ACS Appl. Mater. Interfaces.

[121] (2022). Nanofiber-based biodegradable millirobot with controllable anchoring and adaptive stepwise release functions. Matter.

[122] Ntziachristos V. (2010). Going deeper than microscopy: the optical imaging frontier in biology. Nat. Methods.

[123] Taruttis A., Ntziachristos V. (2015). Advances in real-time multispectral optoacoustic imaging and its applications. Nat. Photonics.

[124] Omar M., Aguirre J., Ntziachristos V. (2019). Optoacoustic mesoscopy for biomedicine. Nat. Biomed. Eng..

[125] Evertsson M. (2017). Combined Magnetomotive ultrasound, PET/CT, and MR imaging of 68Ga-labelled superparamagnetic iron oxide nanoparticles in rat sentinel lymph nodes in vivo. Sci. Rep..

[126] Shin T.-H., Choi Y., Kim S., Cheon J. (2015). Recent advances in magnetic nanoparticle-based multi-modal imaging. Chem. Soc. Rev..

[127] Aziz A. (2020). Medical imaging of microrobots: toward in vivo applications. ACS Nano.

[128] Yan C. (2024). Biohybrid nanorobots carrying Glycoengineered extracellular vesicles promote diabetic wound repair through dual‐enhanced cell and tissue penetration. Adv. Sci..

[129] Xie S. (2022). Self-propelling nanomotors integrated with biofilm microenvironment-activated NO release to accelerate healing of bacteria-infected diabetic wounds. Adv. Healthcare Mater..

[130] Chen L. (2023). Nanomotors-loaded microneedle patches for the treatment of bacterial biofilm-related infections of wound. J. Colloid Interface Sci..

[131] (2024). Magnetically-Enhanced Diffusion (MED (TM)) of Intravenous tPA in Acute Ischemic Stroke: A Pilot Safety and Feasibility Trial | Request PDF.

[132] Dai H. (2022). Stability, aggregation, and sedimentation behaviors of typical nano metal oxide particles in aqueous environment. J. Environ. Manage..

[133] Jiang Z., Fu L., Wei C., Fu Q., Pan S. (2023). Antibacterial micro/nanomotors: advancing biofilm research to support medical applications. J. Nanobiotechnol..

[134] Agrahari V. (2020). Intelligent micro-/nanorobots as drug and cell carrier devices for biomedical therapeutic advancement: promising development opportunities and translational challenges. Biomaterials.

[135] Li J. (2023). Antimicrobial micro/nanorobotic materials design: from passive combat to active therapy. Mater. Sci. Eng. R Rep..

[136] Matai I. (2014). Antibacterial activity and mechanism of Ag–ZnO nanocomposite on *S. aureus* and GFP-expressing antibiotic resistant *E. coli*. Colloids Surf., B Biointerfaces.

[137] Pelgrift R.Y., Friedman A.J. (2013). Nanotechnology as a therapeutic tool to combat microbial resistance. Adv. Drug Deliv. Rev..

[138] Brown A.N. (2012). Nanoparticles functionalized with ampicillin destroy multiple-antibiotic-resistant isolates of Pseudomonas aeruginosa and Enterobacter aerogenes and methicillin-resistant Staphylococcus aureus. Appl. Environ. Microbiol..

[139] Radovic-Moreno A.F. (2012). Surface charge-switching polymeric nanoparticles for bacterial cell wall-targeted delivery of antibiotics. ACS Nano.

[140] Gupta A. (2018). Engineered polymer nanoparticles with unprecedented antimicrobial efficacy and therapeutic Indices against multidrug- resistant bacteria and biofilms. J. Am. Chem. Soc..

[141] Miller K.P. (2015). Engineering nanoparticles to silence bacterial communication. Front. Microbiol..

[142] Makabenta J.M.V. (2021). Nanomaterial-based therapeutics for antibiotic-resistant bacterial infections. Nat. Rev. Microbiol..

[143] Dong Y. (2021). Magnetic microswarm composed of porous nanocatalysts for targeted elimination of biofilm occlusion. ACS Nano.

[144] Wang B., Kostarelos K., Nelson B.J., Zhang L. (2021). Trends in micro-/Nanorobotics: materials development, actuation, localization, and system integration for biomedical applications. Adv. Mater..

